# Quantitative proteomics reveals the protective effects of Yinchenzhufu decoction against cholestatic liver fibrosis in mice by inhibiting the PDGFRβ/PI3K/AKT pathway

**DOI:** 10.3389/fphar.2024.1341020

**Published:** 2024-02-26

**Authors:** Qian Meng, Hongwen Zhu, Yuanyuan Li, Xiaotian Peng, Tianming Wang, Hui Huang, Hu Zhou, Yuejia Liu, Sujie Ru, Jiasheng Wu, Yueming Ma

**Affiliations:** ^1^ Department of Pharmacology, School of Pharmacy, Shanghai University of Traditional Chinese Medicine, Shanghai, China; ^2^ Analytical Research Center for Organic and Biological Molecules, State Key Laboratory of Drug Research, Shanghai Institute of Materia Medica, Chinese Academy of Sciences, Shanghai, China; ^3^ University of Chinese Academy of Sciences, Beijing, China; ^4^ School of Chinese Materia Medica, Nanjing University of Chinese Medicine, Nanjing, Jiangsu, China

**Keywords:** Yinchenzhufu decoction, proteomics, cholestasis, liver fibrosis, PDGFRβ/PI3K/AKT

## Abstract

**Introduction:** Yinchenzhufu decoction (YCZFD) is a traditional Chinese medicine formula with hepatoprotective effects. In this study, the protective effects of YCZFD against cholestatic liver fibrosis (CLF) and its underlying mechanisms were evaluated.

**Methods:** A 3, 5-diethoxycarbonyl-1, 4-dihydro-collidine (DDC)-induced cholestatic mouse model was used to investigate the amelioration of YCZFD on CLF. Data-independent acquisition-based mass spectrometry was performed to investigate proteomic changes in the livers of mice in three groups: control, model, and model treated with high-dose YCZFD. The effects of YCZFD on the expression of key proteins were confirmed in mice and cell models.

**Results:** YCZFD significantly decreased the levels of serum biochemical, liver injury, and fibrosis indicators of cholestatic mice. The proteomics indicated that 460 differentially expressed proteins (DEPs) were identified among control, model, and model treated with high-dose YCZFD groups. Enrichment analyses of these DEPs revealed that YCZFD influenced multiple pathways, including PI3K-Akt, focal adhesion, ECM–receptor interaction, glutathione metabolism, and steroid biosynthesis pathways. The expression of platelet derived growth factor receptor beta (PDGFRβ), a receptor associated with the PI3K/AKT and focal adhesion pathways, was upregulated in the livers of cholestatic mice but downregulated by YCZFD. The effects of YCZFD on the expression of key proteins in the PDGFRβ/PI3K/AKT pathway were further confirmed in mice and transforming growth factor-β-induced hepatic stellate cells. We uncovered seven plant metabolites (chlorogenic acid, scoparone, isoliquiritigenin, glycyrrhetinic acid, formononetin, atractylenolide I, and benzoylaconitine) of YCZFD that may regulate PDGFRβ expression.

**Conclusion:** YCZFD substantially protects against DDC-induced CLF mainly through regulating the PDGFRβ/PI3K/AKT signaling pathway.

## 1 Introduction

Cholestatic liver disease (CLD) is a hepatobiliary disorder characterized by liver damage due to obstructions in bile formation, secretion, and/or excretion. CLD is associated with the accumulation of bile acids (BAs) in the liver, resulting in liver inflammation, hepatocyte damage, and liver fibrosis and cirrhosis, for which treatment options are limited (Goodman, 2007; Gines et al., 2021). Persistent cholestasis may lead to chronic inflammatory responses within the liver and damage bile duct cells and hepatocytes (Cai et al., 2017; Zeng et al., 2023). Various factors associated with hepatocyte apoptosis and necrosis, including inflammation in the liver and activation of Kupffer cells, trigger the secretion of pro-inflammatory cytokines (Kisseleva and Brenner, 2021). Alongside various chemical messengers, these cytokines activate and transform hepatic stellate cells (HSCs) into myofibroblasts. Furthermore, activated HSCs can enhance myofibroblast proliferation through paracrine or autocrine mechanisms, resulting in the synthesis of abundant collagen fibers and other extracellular matrix (ECM) components. During this process, regulatory factors such as platelet-derived growth factor (PDGF) can interact with the ECM in a complex network to promote liver fibrogenesis.

Cholestatic liver fibrosis (CLF) is a serious pathological process in CLD development (Novo et al., 2009). Ursodeoxycholic acid (UDCA) is an FDA-approved drug for the treatment of primary biliary cholangitis (PBC) (Lindor, 2007). However, UDCA is often unavailable or intolerable. Alternatively, obeticholic acid (OCA) is used for patients with UDCA intolerance, but its application is limited by various adverse reactions, such as severe pruritus (Beuers et al., 2015). Moreover, the efficacies of both agents in primary sclerosing cholangitis (PSC) remain unclear (Ghonem et al., 2015). As a result, limited drugs to treat cholestasis and liver fibrosis are available.

Traditional Chinese medicine (TCM) formulas exert therapeutic effects against cholestasis via multiple signaling pathways, including pathways related to bile acid metabolism, gut microbiota, inflammation, and fibrosis (Wei et al., 2022). In TCM, CLD belongs to the category of jaundice, which can be divided into Yin-yellow and Yang-yellow syndromes. Yinchenzhufu decoction (YCZFD) is a classical TCM formula for the treatment of Yin-yellow syndrome. YCZFD consists of six herbs: Artemisiae Scopariae Herba (*Artemisia capillaris* Thunb.), Atractylodis Macrocephalae Rhizoma (*Atractylodes macrocephala* Koidz.), Aconiti Lateralis Radix Praeparaia (*Aconitum carmichaelii* Debx.), Zingiberis Rhizoma (*Zingiber officinale* Rosc.), Glycyrrhizae Radix et Rhizoma Praeparata (*Glycyrrhiza uralensis* Fisch.), and Cinnamomi Cortex (*Cinnamomum cassia* Presl.). Previous studies of acute cholestatic model induced by alpha-naphthylisothiocyanate and chronic cholestatic mouse model induced by 3,5-diethoxycarbonyl-1,4-dihydroxychollidine (DDC) found that YCZFD exerts a hepatoprotective effect through ameliorating disordered BAs homeostasis and inflammation (Wang et al., 2020; Li et al., 2023). YCZFD has been widely used for treating the Yin-yellow syndrome, which belongs to chronic liver disease, in clinical settings (Zhu Fanghong and Li, 2021). However, whether YCZFD protects against CLF and its molecular mechanisms are unclear.

Quantitative proteomic analysis has been widely employed to investigate the formation and progression of cholestasis, identify disease biomarkers, and find the alterations in protein and signaling pathways under drug intervention, thereby providing preliminary insights into the characteristic biomarkers and pathobiology of diseases such as PSC and PBC (Massafra et al., 2017; Li et al., 2019b; Wu et al., 2020; Wang et al., 2021a). Previous studies have explored the novel mechanisms of TCM formulas at the protein level (Lao et al., 2014). In the present study, quantitative proteomics was used to elucidate the potential networks modulated by YCZFD in DDC-induced cholestatic mice. Our results indicated that YCZFD ameliorates CLF mainly via the PDGFRβ/PI3K/AKT signaling pathway.

## 2 Materials and methods

### 2.1 Materials

The chemicals and reagents are described in Supplementary Material M1. Crude drugs *Artemisia capillaris* Thunb*.* (210218), *Atractylodes macrocephala* Koidz. (210430), *Aconitum carmichaelii* Debx. (210309), *Zingiber officinale* Rosc. (210403), *Glycyrrhiza uralensis* Fisch. (2106034), and *Cinnamomum cassia* Presl. (210309) were purchased from Shanghai Kangqiao Chinese Medicine Tablet Co., Ltd. and authenticated using morphological and microscopic identification in accordance with the Chinese Pharmacopoeia by Dr. Jinrong Wu from Shanghai University of Traditional Chinese Medicine (Commission, 2020).

### 2.2 YCZFD preparation and quality control

YCZFD extract was prepared according to our previously reported method (Wang et al., 2020). Briefly, the crude materials of *Artemisia capillaris* Thunb. (300 g), *Atractylodes macrocephala* Koidz. (600 g), *Aconitum carmichaelii* Debx. (150 g), *Zingiber officinale* Rosc. (150 g), *Glycyrrhiza uralensis* Fisch. (300 g), and *Cinnamomum cassia* Presl. (100 g) were immersed in 16 L of water for 30 min and boiled for 1 h to collect the first decoction. After filtration, 12.8 L of water was added and the mixture was boiled for 1 h to collect the second decoction. The two decoctions were mixed and concentrated with a rotary evaporator. The mixture was freeze-dried to obtain powdered YCZFD extract with 19% yield.

Qualitative analysis using an ultra-high performance liquid chromatography coupled to a linear trap quadrupole-Orbitrap (UHPLC-LTQ-Orbitrap) elite MS system (Thermo Fisher Scientific, Bremen, Germany) (Wang et al., 2020) revealed 58 plant metabolites in YCZFD extract (Supplementary Table S1). The quality control of YCZFD was performed as the methods described previously (Li et al., 2023). A total of 18 chemical plant metabolites (chlorogenic acid, scoparone, 4-hydroxyacetophenone, atractylenolide I, atractylenolide Ⅱ, atractylenolide Ⅲ, benzoylaconine, benzoylhypaconine, benzoylmesaconitine, formononetin, glycyrrhitinic acid, glycyrrhizic acid, isoliquiritigenin, isoliquiritin, liquiritigenin, liquiritin, ononin, and cinnamic acid) were quantified and shown in Supplementary Table S2. Among these plant metabolites, glycyrrhizic acid (1275.88 μg/g), liquiritin (901.26 μg/g), chlorogenic acid (782.2 μg/g), isoliquiritin (91.1 μg/g), and atractylenolide I (39.11 μg/g) had the highest contents.

### 2.3 Animal experiment

**FIGURE 1:**
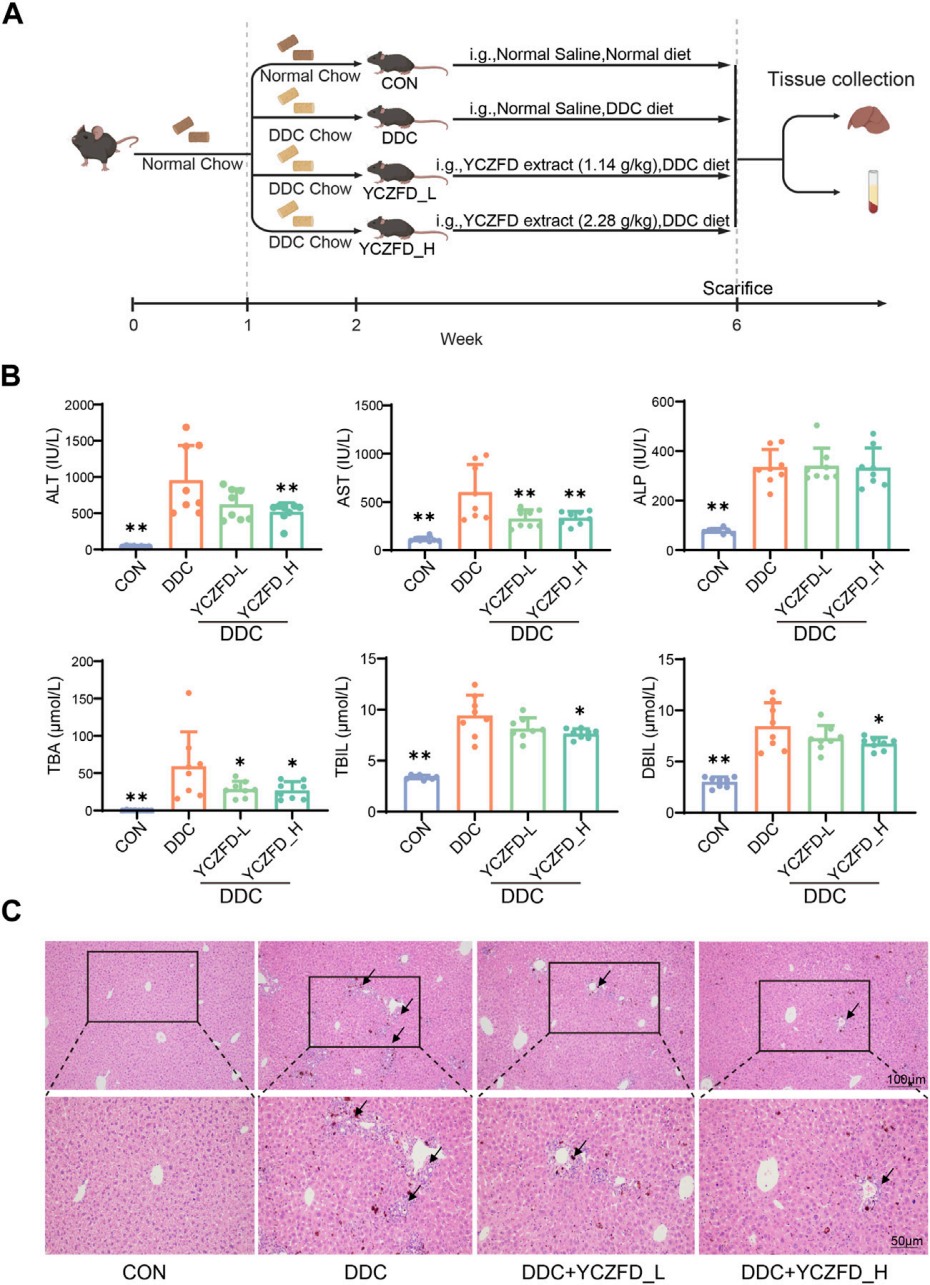
YCZFD protects against 3,5-diethoxycarbonyl-1,4-dihydroxychollidine (DDC)-induced intrahepatic cholestasis in mice. **(A)** Scheme of animal experiment; **(B)** Biochemical analysis of serum samples from each group; **(C)** Histological analyses of liver tissues under different conditions (H&E, scale bar 100 μm and 50 μm). Data are presented as means ± SD, *n* = 8 for serum biochemical parameters and histological analyses; **p < 0.01 and *p < 0.05 compared with the DDC group. ALT, alanine aminotransferase; AST, aspartate transaminase; ALP, alkaline phosphatase; TBA, total bile acid; TBIL, total bilirubin; DBIL, direct bilirubin.

Male C57BL/6J mice weighing 20 ± 2 g were obtained from Shanghai SLAC Laboratory Animal Co., Ltd. The mice were housed and acclimatized for 1 week at the Animal Experimental Center of Shanghai University of Traditional Chinese Medicine (SHUTCM). The temperature and humidity in the breeding room were maintained at 20°C–26°C and 40%–60%, respectively. Pharmaceutical experiments were performed after 1 week. The mice were divided into four groups (n = 8): control (CON), DDC model (DDC), DDC+YCZFD-L (1.14 g/kg YCZFD extract, equivalent to 6 g crude drug/kg), and DDC+YCZFD-H (2.28 g/kg YCZFD extract, equivalent to 12 g crude drug/kg). The mice in the CON group were fed a normal diet, while those in the three other groups were fed a diet containing 0.025% DDC. After 1 week of diet feeding, the mice in the CON and DDC groups were orally administered normal saline, while those in the DDC+YCZFD-L and DDC+YCZFD-H groups were orally treated with YCZFD extract once a day for the following 4 weeks. During the drug treatment period, the mice in the DDC, DDC+YCZFD-L, and DDC+YCZFD-H groups were still fed a diet containing 0.025% DDC. The experimental design is shown in Figure 1A. All mice were sacrificed at the experimental endpoint, and the serum and liver tissues were collected for further studies. Animal experiments were approved by the Experimental Animal Welfare and Ethics Committee of SHUTCM (Approval number Pzshutcm190823002).

### 2.4 Serum biochemistry and histological analysis

Details regarding serum biochemistry and liver histopathological morphology analysis can be found in Supplementary Material M2.

### 2.5 Immunohistochemistry (IHC)

Mouse livers fixed by formalin were paraffin embedded, sectioned, and then stained with an anti-alpha-smooth muscle actin (anti-α-SMA) or collagen type 1 alpha 1 chain (COL1A1) antibody sourced from Abcam (Cambridge, MA, United States). To facilitate histological examination, the sections were ultimately affixed using the DPX Mountant (Sigma).

### 2.6 Quantitative proteomics analysis

#### 2.6.1 Protein extraction and peptide digestion

Liver tissue samples from the mice in the CON, DDC, and YCZFD-H groups (8 mice per group) were washed with phosphate buffered saline (PBS) to remove residual blood on the tissue surface. Subsequently, the samples were lysed with an SDT buffer composed of 4% sodium dodecyl sulfate, 100 mM dithiothreitol, and 100 mM Tris (pH 7.6). The samples were crushed using a tissue homogenizer, sonicated, and then heated at 95°C. After centrifugation, the supernatant was collected, and the protein concentration was determined using tryptophan-based fluorescence quantification (Thakur et al., 2011). Peptides were generated in accordance with the Filter Assisted Sample Preparation protocol as detailed in Supplementary Material M3 (Wisniewski et al., 2009).

#### 2.6.2 Mass spectrometry

The Thermo Fisher nLC1000 HPLC system and Thermo Fisher Q Exactive HF mass spectrometer were used for LC-MS/MS. A self-packed separation column was used (75 μm × 200 mm, 3.0 µm ReproSil-Pur 120 C18-AQ resin). Mobile phase A was 100% H_2_O containing 0.1% formic acid, and mobile phase B was 100% acetonitrile containing 0.1% formic acid. The peptide sample (1 µg) was injected for LC-MS/MS. The peptides were detected in data-independent acquisition (DIA) mode. The chromatographic gradient was set to 90 min with the following settings: 1%–5% B for 0–1 min, 5%–26% B for 1–75 min, 26%–32% B for 75–83 min, 32%–90% B for 83–85 min, and 90% B for 85–90 min. The DIA parameters were set as follows: scan range, 350–1,600 Da; first-level mass resolution, 120,000; AGC target, 3e6; and maximum injection time, 20 ms. Data were collected using 40 variable windows. The second-level mass resolution was set at 30,000, AGC target at 5e5, and normalized collision energy at 27.

DIA data were analyzed using Spectronaut software (version 14, Biognosys). The search parameters were set as follows: for quantification, major and minor group quantities were set as sum peptide quantity and sum precursor quantity, respectively, and local normalization was applied. Other parameters were set to default. After the database search, the raw data and the data with quantificative information were exported for subsequent analyses.

#### 2.6.3 Data processing and analysis

Data processing and analysis were performed using R. Proteins that were quantified in more than 60% of the samples were retained, and column data were median normalized. Comparisons were performed using one-way analysis of variance (ANOVA), and the Benjamini–Hochberg (BH) procedure was applied to correct for multiple *p*-values. The criteria for selecting differentially expressed proteins (DEPs) were as follows: BH-adjusted *p*-value < 0.05 and fold change (FC) ≥ 1.2 or FC ≤ 1/1.2. Fuzzy c-means clustering was performed using the Mfuzz package in R. Functional enrichment of Gene Ontology (GO) biological process and Kyoto Encyclopedia of Genes and Genomes (KEGG) were performed using the DAVID database (https://david.ncifcrf.gov/), and visualization was conducted in R. The STRING database was used to predict protein–protein interaction (PPI) networks, and Cytoscape (version 3.6.1) was used for visualization of PPI networks.

### 2.7 Hepatic stellate cell culture and treatment

The human HSC line (LX-2) was cultured in Dulbecco’s modified Eagle’s medium (DMEM) containing 10% FBS and incubated at 37°C in an atmosphere of 5% CO_2_. Cells were treated with 5 ng/mL transforming growth factor-β (TGF-β) or TGF-β (5 ng/mL) and increasing concentrations (5, 10, 25, and 50 μg/mL) of YCZFD for 48 h.

### 2.8 Real-time PCR

Details regarding real-time PCR are provided in Supplementary Material M4 and Supplementary Table S2.

### 2.9 Western blot

Details regarding Western blot analysis are presented in Supplementary Material M5. The primary antibodies were as follows: anti-FN1 (151613-1-AP, Proteintech), anti-COL1A1 (NBP1-30054, Novus), anti-α-SMA (ab124964, Abcam), anti-TIMP2 (5738, CST), anti-TGF-β (ab92486, Abcam), anti-GAPDH (G8795, Sigma-Aldrich), anti-PDGFRβ (13449-1-AP, Proteintech), anti-p-PDGFRβ (AP0815, ABclonal), anti-PDGF-B (ab178409, Abcam), anti-AKT1(2967S, CST), anti-p-AKT1 (15116, CST), anti-PI3K (19H8, CST), anti-p-PI3K (AT-3241, Affinity), and anti-proliferating cell nuclear antigen (anti-PCNA; 61079, Active motif) antibodies.

### 2.10 Cell cycle assay

LX-2 were cultured in DMEM with TGF-β (5 ng/mL), or with TGF-β (5 ng/mL) and YCZFD extract (5 and 25 μg/mL) for 48 h. Subsequently, the cells were harvested in cold PBS at 4°C and then fixed using 70% ethanol at the same temperature overnight. Following fixation, the cells were washed with cold PBS to remove excess fixative and then stained with propidium iodide containing RNase A. FlowJo (Tree Star) was employed to evaluate the DNA content of the stained cells.

### 2.11 Extraction and cultivation of primary hepatocytes

After the injection of 25% pentobarbital, incision of abdominal wall, and separation of the portal vein, a cannula connected to an infusion tube was inserted into the portal vein, the inferior vena cava was cut, and a peristaltic pump was turned on. Perfusion solution was perfused for 40 min. The liver was placed in culture medium and then agitated to release hepatocytes. The extracted cells were passed through a 200-mesh cell strainer, centrifuged, and then resuspended in DMEM. The cells were transferred to the upper layer of the medium containing Percoll and then centrifuged. The viable cells were resuspended in DMEM, centrifuged, resuspended in Williams’ MediumE (WME) containing 10% FBS, 10 nM insulin, and 10 nM dexamethasone, and counted.

On the day before the sandwich culture and treatment of primary cells, the lower gelatin layer [100 mL of ultrapure water + 114 μL of ice-cold acetic acid (0.02 M) + 1.6 mL of type I mouse tail collagen] was prepared, and the culture dishes were coated. On the day of primary cell extraction, the plates were seeded. On the second day, the upper gelatin layer (50 mL system: 2.5 mL of serum, 0.5 mL of glutamine, 0.5 mL of dexamethasone, 0.5 mL of ITS, and 46.45 mL of WME) was prepared. On the third day, drug treatment was administered. The CCK-8 assay was used to screen the safe dosage range of plant metabolites of YCZFD (Supplementary Figure S2). Glycyrrhetinic acid at 10 μM and the other plant metabolites at 30 μM did not affect the viability of primary cells and thus were used in subsequent experiments. Taurocholate acid (TCA) and TCA combined with individual plant metabolites were administered simultaneously. After 24 h of incubation, the mRNA expression of *PDGFRβ* was detected.

### 2.12 Statistical analysis

The data were statistically analyzed using GraphPad Prism version 8.0 (GraphPad Software, La Jolla, CA, United States). ANOVA followed by Tukey *post hoc* tests was performed. Data are presented as mean ± SD. *p* < 0.05 was considered statistically significant.

## 3 Results

### 3.1 YCZFD ameliorates cholestatic liver injury (CLI) in DDC-induced mice

After treatment with YCZFD, the DDC-induced increases in ALT, AST, TBA, DBIL, and TBIL were significantly attenuated (Figure 1B). Histopathological assessments revealed severe hepatic necrosis, inflammatory cell infiltration, and bile duct proliferation in the liver tissues of the mice subjected to DDC intervention. Treatment with YCZFD decreased the liver damage (Figure 1C). Overall, these findings demonstrated that treatment with YCZFD ameliorated CLI in the DDC**-**induced mice.

### 3.2 YCZFD alleviates CLF in DDC-induced mice

**FIGURE 2:**
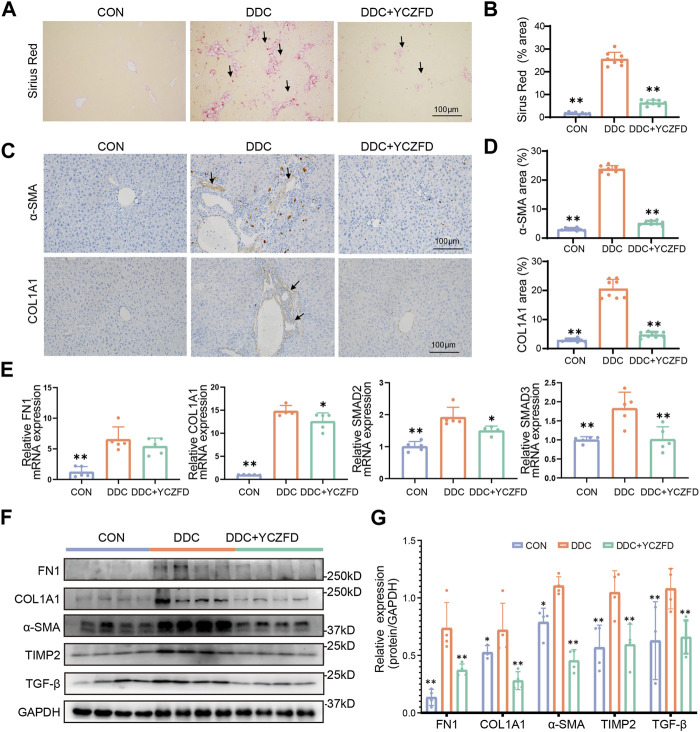
YCZFD protects against DDC-induced cholestatic liver fibrosis in mice. **(A)** Sirius red-stained liver sections under Control (CON), DDC-induced model (DDC), and DDC combined with high-dose YCZFD treatment (DDC+YCZFD) groups (*n* = 8) (scale bar, 100 μm). **(B)** Quantification of Sirius red staining for each group is shown in a bar graph (Ten random fields were taken in one slide and scoring by a blinded experimenter). **(C)** Representative images of immunohistochemical staining of α-SMA and COL1A1 in each group (*n* = 8) (scale bar, 100 μm). **(D)** Quantification of α-SMA and COL1A1 staining for each group is shown in a bar graph (Ten random fields were taken in one slide and scoring by a blinded experimenter). **(E)** mRNA expression of COL1A1, FN1, SMAD2, and SMAD3 in mouse liver tissues in each group (*n* = 5). **(F)** Liver protein expression of FN1, COL1A1, α-SMA, TIMP2, and TGF-β in each group detected using Western blot. (*n* = 4 per group). **(G)** Quantification of Western blot data in **(F)**. Data are presented as means ± SD; **p < 0.01 and *p < 0.05 compared with the DDC group.

Sirius red staining showed greater parenchymal matrix deposition within the liver tissues in the DDC group than that in the CON group (Figures 2A, B), and this increase in collagen deposition was attenuated in the livers of the YCZFD treatment group. Immunohistochemical staining revealed that the increased expression of liver fibrosis markers (α-SMA and COL1A1) in the fibrotic septa of the DDC group was decreased in the YCZFD treatment group (Figures 2C, D). Consistent with the immunohistochemical staining results, the protein expression of COL1A1, FN1, and α-SMA and the mRNA expression of *COL1A1* were increased in the DDC group and attenuated in the YCZFD group (Figures 2E–G). Additionally, the gene expression levels of *SMAD2* and *SMAD3* and the protein levels of TGF-β in the cholestatic mice were significantly decreased by YCZFD treatment. Overall, these findings demonstrated that treatment with YCZFD ameliorated CLF.

### 3.3 Identification and quantification of differentially expressed proteins (DEPs) influenced by YCZFD

**FIGURE 3:**
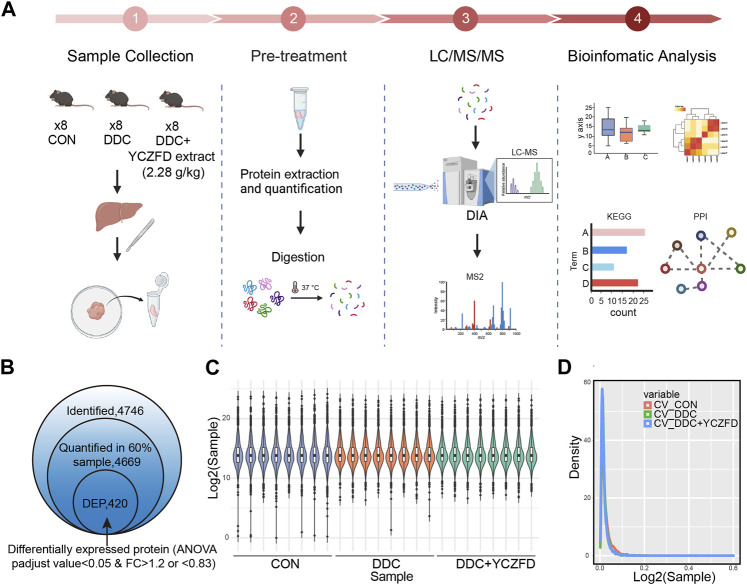
Proteomic analysis of mouse liver tissues from the CON, DDC, and DDC+YCZFD groups. **(A)** Workflow chart of label-free quantitative proteomic experiment (*n* = 8 for each group). **(B)** Summary of identified and quantified proteins. In total, 420 proteins were differently expressed in the YCZFD treatment and CON groups compared with the DDC group, respectively. **(C)** Boxplot of protein profiles. **(D)** Density distribution of coefficient of variation in three groups.

DIA technology was used for the quantitative proteomic analysis of liver tissues from the CON, DDC, and DDC+YCZFD (DDC+YCZFD-H) groups to elucidate the mechanism by which YCZFD ameliorates cholestatic liver fibrosis. The experimental workflow is shown in Figure 3A. A total of 4,746 proteins were identified, and 4,669 proteins had quantitative information in 60% of the samples (Figure 3B). The proteomic data were subjected to median normalization for each sample to eliminate variation in sample injection amounts (Figure 3C). The distribution of protein abundance in each sample was similar, indicating good parallelism among samples. Furthermore, the median coefficients of variation among the eight replicate samples from the CON, DDC, and YCZFD groups were all less than 0.1 (Figure 3D), indicating good quantitative reproducibility of the data. Overall, the proteomic data were of high quality. ANOVA was used to identify DEPs among the three groups. Based on BH-corrected *p-*values (*p* < 0.05) and fold change (>1.2 or <0.83), 420 DEPs were identified (Supplementary Table S3).

### 3.4 Enrichment analysis for DEPs

**FIGURE 4:**
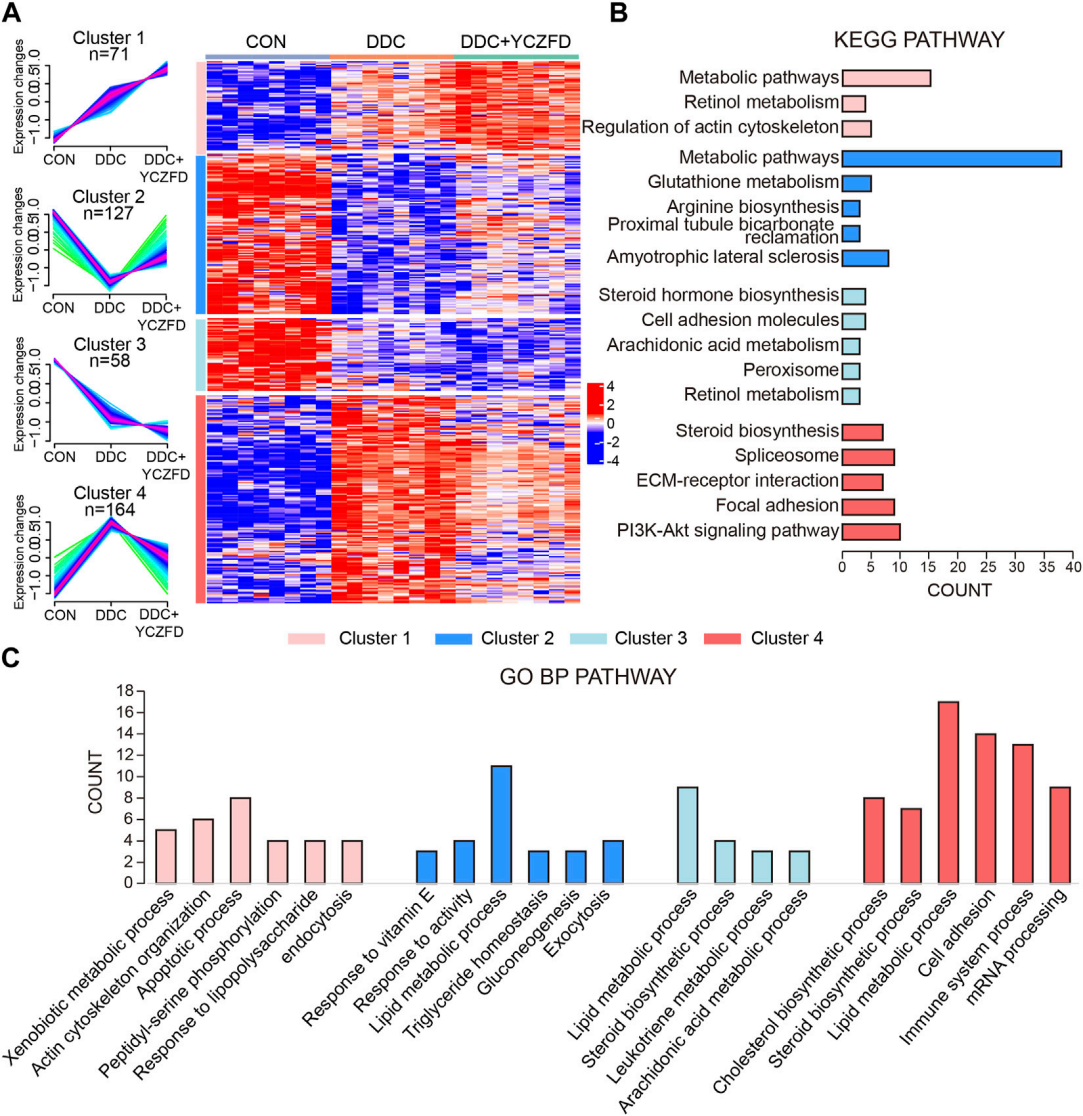
Identification of dynamic expression patterns and molecular characteristics of 420 differential proteins. **(A)** Differentially regulated proteins were separated into four clusters through the fuzzy c-means soft clustering method (left panel) and HCA (right panel). **(B)** KEGG analysis of the proteins in each cluster. **(C)** GO analysis of biological processes of the proteins in each cluster.

The DEPs were clustered into four subclusters (Figure 4A, left panel) by using the mfuzz clustering algorithm, and a pheatmap was used to visualize the expression levels (Figure 4A, right panel). Proteins in Cluster 2 and Cluster 4 were differentially expressed between the DDC and CON groups, with the attenuation of expression changes after YCZFD intervention. Cluster 2 consisted of 127 proteins, with downregulated expression in the DDC group and restored expression after YCZFD treatment (Figure 4A). Cluster 4 consisted of 164 proteins, with upregulated expression in the DDC group and restored expression after YCZFD treatment.

Proteins in the four clusters were evaluated through GO biological process and KEGG pathway enrichment analyses (Figures 4B, C). Proteins in Cluster 2 were mainly enriched in glutathione metabolism, proximal tubule bicarbonate reabsorption pathways, and lipid metabolic process. Proteins in Cluster 4 were mainly enriched in the sterol biosynthesis pathway, spliceosome signaling pathways, ECM–receptor interaction, PI3K-AKT pathway, and cell adhesion process.

### 3.5 Enrichment of PDGFRβ in cholestatic by a bioinformatics analysis

**FIGURE 5:**
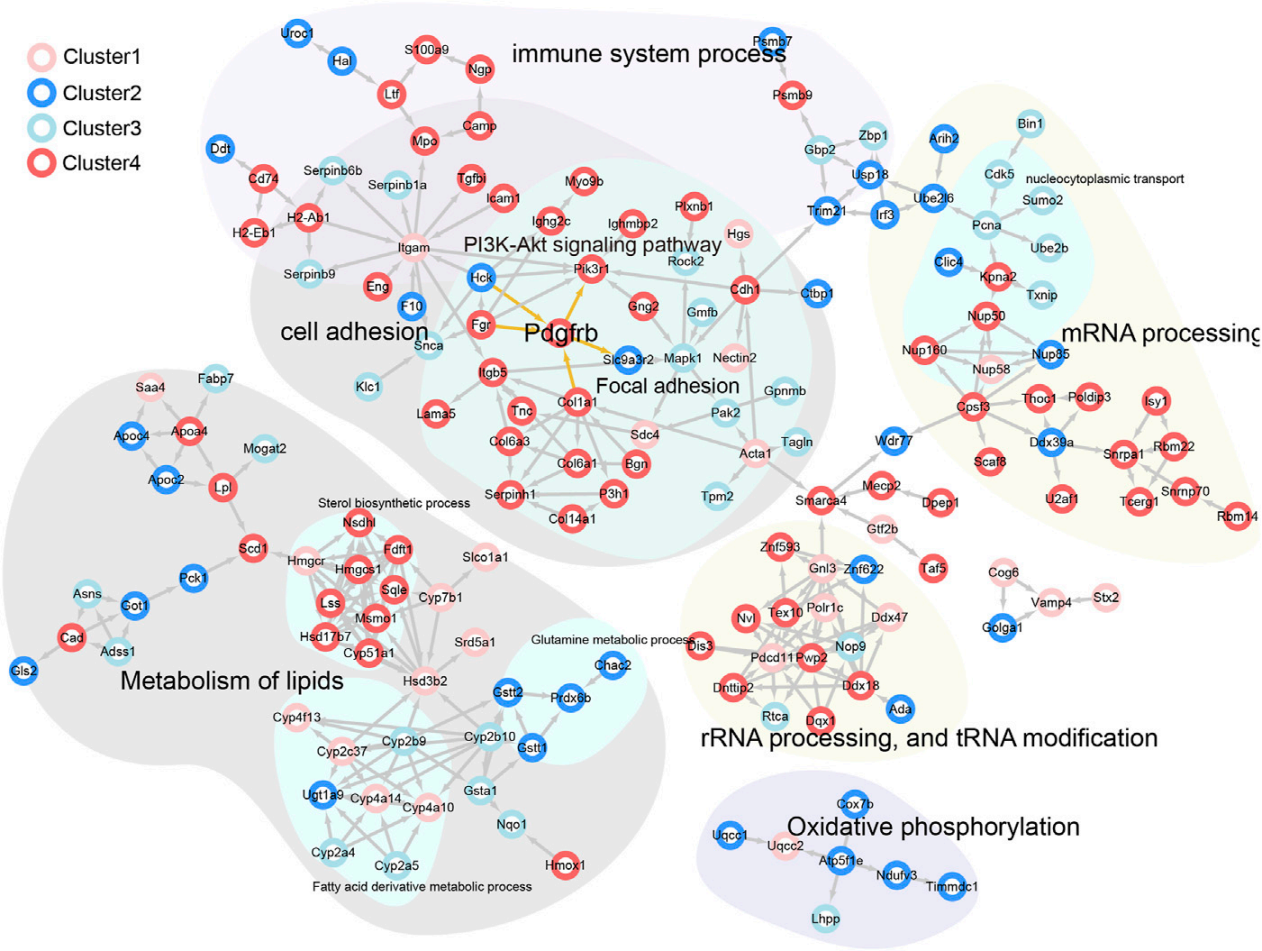
PPI network analysis of potential interacting proteins corresponded to different clusters in the above biological processes and pathways.

The PPI network and pathway enrichment results were integrated (Figure 5). Pathways related to sterol metabolism were significantly enriched, including sterol synthesis-related proteins (e.g., Sqle, Hmgcs1, and Nsdh1), which belong to Cluster 4 (Supplementary Figure S1). Importantly, PI3K-AKT and focal adhesion-related proteins, such as PDGFRβ, PIKR3R1, ITGB5, COL1A1, COL6A1, COL6A3, and LAMA5, are mainly associated with liver fibrosis. PDGFRβ was identified as a hub protein, suggesting that YCZFD exerts a protective effect against cholestasis by alleviating fibrosis through the regulation of PDGFRβ.

### 3.6 YCZFD regulates PDGFRβ/PI3K/AKT in the fibrotic liver of cholestatic mice

**FIGURE 6:**
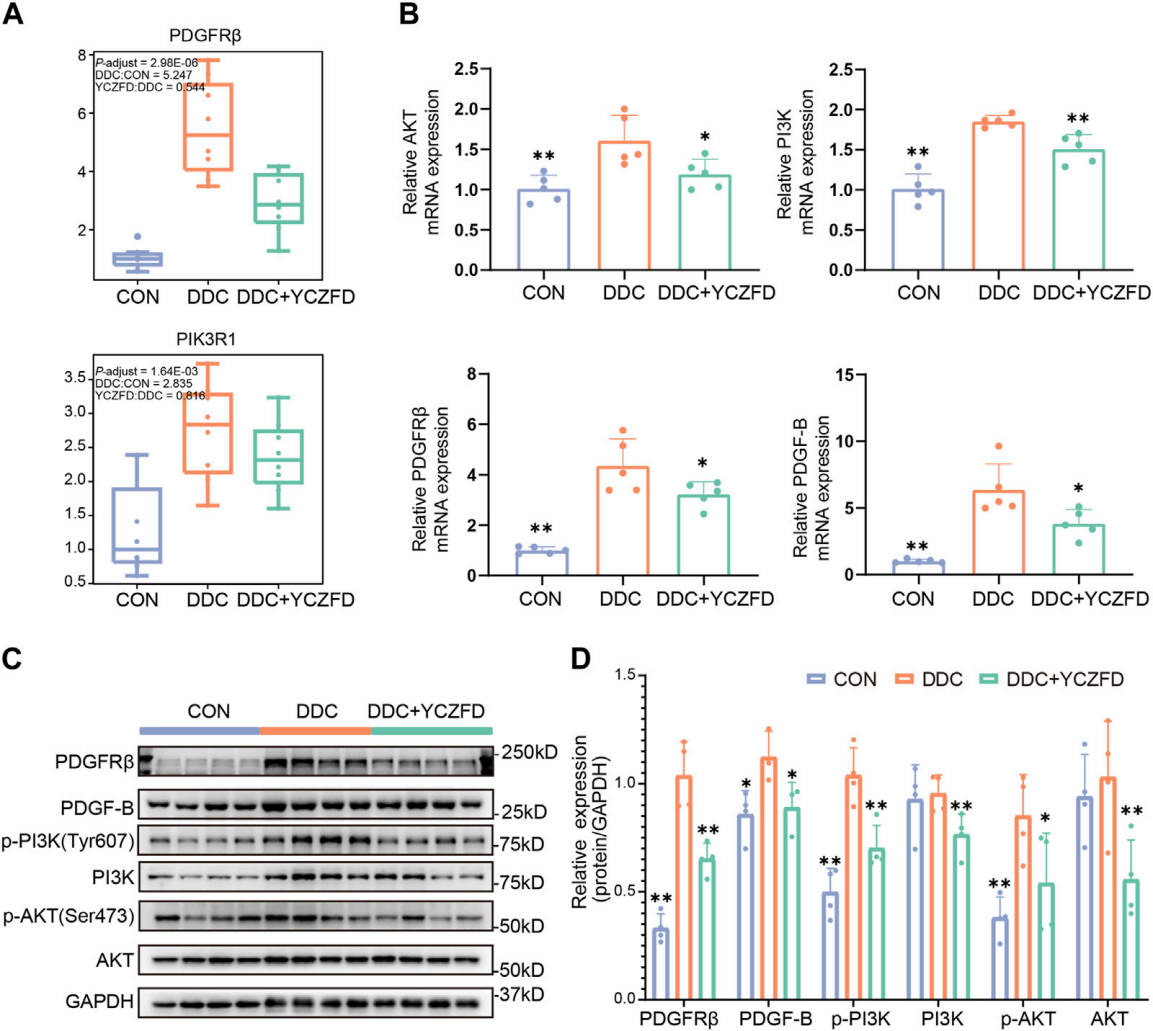
YCZFD regulates the PDGFRβ-PI3K-Akt pathway anti-liver fibrosis. **(A)** Boxplot of the relative abundances of the PDGFRβ and PI3K proteins in each group (*n* = 8 per group). **(B)** RT-PCR results of the mRNA expression of AKT, PI3K, PDGFRβ, and PDGF-B in mouse liver tissues from each group (*n* = 5 per group). **(C)** Liver protein expression of PDGFRβ, p-AKT, AKT, p-PI3K, PI3K, and PDGF-B detected using Western blot (*n* = 4 per group). GAPDH was used as the loading control. **(D)** Quantification of Western blot data in **(C)**. Data are presented as means ± SD; **p < 0.01 and *p < 0.05 compared with the DDC group.

PDGFRβ is a key protein involved in the PI3K/AKT signaling and focal adhesion pathways (Figure 5). As revealed from the proteomic analysis, the protein levels of PDGFRβ were significantly upregulated in the DDC group (fold change > 4) and downregulated after YCZFD administration (fold change > 2) (Figure 6A). Western blot and real-time qPCR results further confirmed these changes (Figures 6B–D). Treatment with YCZFD also downregulated PDGFRβ protein expression in the liver tissues. The expression of the ligand of PDGFRβ, PDGF-B, was also downregulated after YCZFD intervention (Figures 6B–D). The expression of PIK3R1, a regulatory subunit of phosphoinositide 3-kinases (PI3Ks), was significantly upregulated in the DDC group and restored in the YCZFD treatment group (Figure 6A). The activation of PI3K and AKT1 in the DDC group and attenuation in the YCZFD group were validated through Western blot (Figures 6C, D).

### 3.7 YCZFD regulates the PDGFRβ/PI3K/AKT pathway in LX2 cells

**FIGURE 7:**
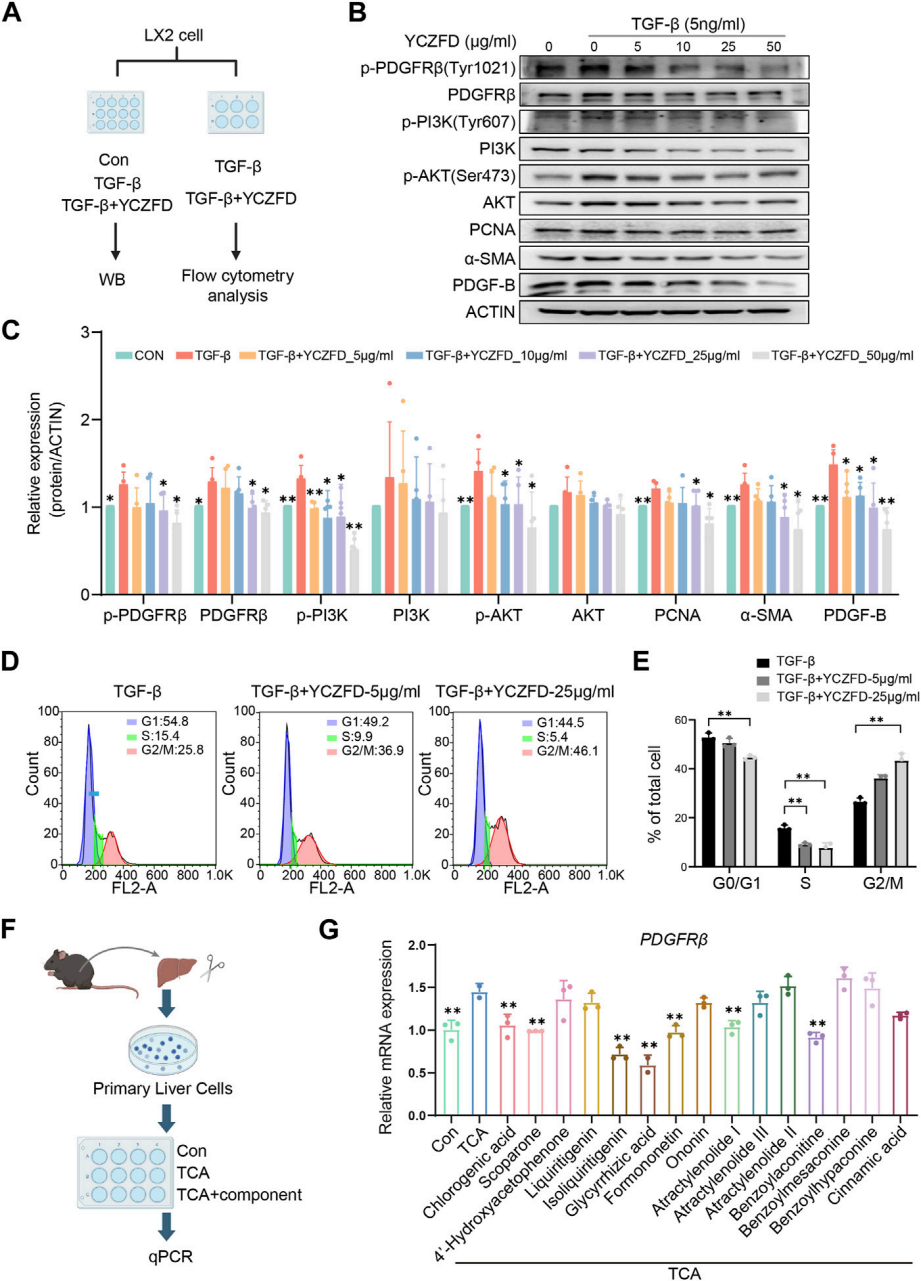
YCZFD regulates the PDGFRB-PI3K-Akt pathway in the human HSC cell line LX-2 and plant metabolites of YCZFD decrease PDGFRβ expression in mouse primary hepatocytes. **(A)** LX-2 were treated with or without 5 ng/mL TGF-β and then stimulated with YCZFD (0, 5, 10, 25, 50 μg/mL) for 48 h. **(B)** The levels of proteins involved in PDGFRβ/PI3K/AKT pathway were measured using Western blot. The experiment was performed five times. **(C)** Quantification of Western blot data in **(B)**. Data are presented as means ± SD; **p < 0.01 and *p < 0.05 compared with the TGF-β group. **(D)** Flow cytometry of TGF-β and TGF-β+YCZFD group cell cycles. **(E)** Quantified flow cytometry results, indicating the proportion of cells in the G1, S, and G2/M phases of the cell cycle. Data are presented as means ± SD. **p < 0.01 and *p < 0.05. **(F)** Workflow chart of identifying active plant metabolites. **(G)** Influence of plant metabolites on the mRNA expression of PDGFRβ. Data are presented as means ± SD; **p < 0.01 and *p < 0.05 compared with the TCA group.

We validated the impact of YCZFD on the PDGFRβ-PI3K-AKT pathway in HSCs (Figure 7A). The expression of α-SMA, which reflects the activated state of HSCs, was increased after TGF-β intervention (Figures 7B, C). The phosphorylation levels of PDGFRβ (Tyr1021), PI3K (Tyr607), and AKT (Ser473) and the protein levels of PDGFRβ and PDGF-B increased in response to TGF-β (Figures 7B, C). Treatment with YCZFD attenuated the changes in levels of these factors. The PCNA expression and cell cycle progression were further investigated. The findings indicated that PCNA expression was downregulated following YCZFD intervention. The composition and ratio of cells in various phases varied with the concentration of YCZFD (0, 5, and 25 μg/mL). Compared to the distribution in TGF-β-treated cells, the percentage of cells in the G2/M phase increased from 25.8% to 36.9% (5 μg/mL YCZFD) and 46.1% (25 μg/mL YCZFD) (Figures 7D, E). These results demonstrated that YCZFD could effectively induce cell cycle arrest in the G2/M phase.

### 3.8 Plant metabolites of YCZFD decrease PDGFRβ expression in mouse primary hepatocytes

To find the effective plant metabolites of YCZFD in primary cells, we utilized a sandwich culture of primary cells incubated them with TCA (known to accumulate in cholestasis) (Figure 7F). PDGFRβ expression was upregulated in the TCA model group compared with the control group (Figure 7G). However, treatment with YCZFD plant metabolites such as chlorogenic acid, scoparone, isoliquiritigenin, glycyrrhetinic acid, formononetin, atractylenolide I, and benzoylaconitine downregulated the mRNA expression of *PDGFRβ* (Figure 7G). These results revealed that multiple plant metabolites of YCZFD may target PDGFRβ.

## 4 Discussion

YCZFD is a representative TCM formula used to treat the Yin-yellow syndrome of jaundice, which is characteristic of chronic CLD and liver fibrosis. Before this study, the effects of YCZFD against CLF and its mechanisms have not been elucidated. The DDC-induced mouse model is a classic chronic cholestatic animal model (Fickert et al., 2007; Li et al., 2021). The main features of this model are bile duct reaction, hepatic inflammation, and fibrosis (Arino et al., 2023; Zhang et al., 2023). In our previous studies, the blood biochemical indicators and liver tissue pathomorphology of mice induced by diet containing 0.025% DDC at 2, 4, 6, and 8 weeks were investigated (Li et al., 2021). Our results confirmed that the liver tissues of mice induced by diet containing 0.025% DDC for 4 weeks have obvious bile duct hyperplasia, collagen deposition, and HSC activation, indicating chronic liver fibrosis (Li et al., 2021). Therefore, in the current study, diet containing 0.025% DDC was used to establish a CLF mouse model. In this study, YCZFD treatment ameliorated the DDC-induced cholestatic liver fibrosis. YCZFD is a classical traditional Chinese herbal prescription that has been used in clinic since the Qing Dynasty in China. The given dose of YZCFD extract (1.14 g/kg, equivalent to 6 g crude) is twice the equivalent dose for clinical use. In our previous animal studies, we found that treatment with YCZFD extract at low, middle, and high doses of 0.57, 1.14, and 2.28 g/kg body weight, respectively, can significantly ameliorate liver injury in cholestatic mice (Wang et al., 2020; Li et al., 2023). Moreover, our results showed that middle- and high-dose YCZFD are more effective than low-dose YCZFD in ameliorating the liver pathological morphology of cholestatic mice (Wang et al., 2020; Li et al., 2023). Therefore, middle- and high-dose YCZFD were used in the present study. Our previous study showed that administering high-dose YCZFD (2.28 g/kg YCZFD extract, equivalent to 12 g crude drug/kg body weight) does not affect the body weight, blood biochemistry, and liver pathology of normal mice (Li et al., 2023), indicating the safety of YCZFD.

To clarify the mechanism of action of YCZFD, we evaluated protein expression in liver tissue samples by using label-free quantitative proteomics technology. The expression levels of 291 proteins belonging to Cluster 2 and Cluster 4 were altered due to DDC injury, and these alterations were reversed by YCZFD treatment, suggesting that these loci are potential targets of YCZFD. In functional enrichment analyses of the DEPs, PI3K-AKT pathway and cell adhesion process were potentially regulated by YCZFD. Previous studies have shown that the PI3K/AKT pathway participates in the formation of liver fibrosis by promoting cell proliferation and collagen synthesis (Zhang et al., 2019; Gong et al., 2020). The focal adhesion complex provides a direct sensor for the integrity of the extracellular environment (Zhang et al., 2019). Moreover, PPI and pathway enrichment analyses revealed that PDGFRβ is a hub protein that interacts with PI3K-AKT and focal adhesion-related proteins. The expression of PDGFRβ, a receptor for PDGFs, was significantly upregulated in the liver of cholestatic mice induced by DDC and downregulated by YCZFD treatment. In the liver fibrosis mouse model, PDGFRβ expression is upregulated in activated HSCs (Reichenbach et al., 2012; Klinkhammer et al., 2018; Zhang et al., 2019; Wang et al., 2021b). The binding of PDGFRβ to its ligands, such as PDGF-B, can activate the downstream PI3K/AKT pathway and promote the proliferation and activation of HSCs (Kinnman et al., 2001; Krampert et al., 2008; Reichenbach et al., 2012; Klinkhammer et al., 2018; Zhang et al., 2019; Wang et al., 2021b). Our current proteomic results suggested that YCZFD protected against CLF by regulating the PDGFRβ**/**PI3K/AKT pathway.

**FIGURE 8:**
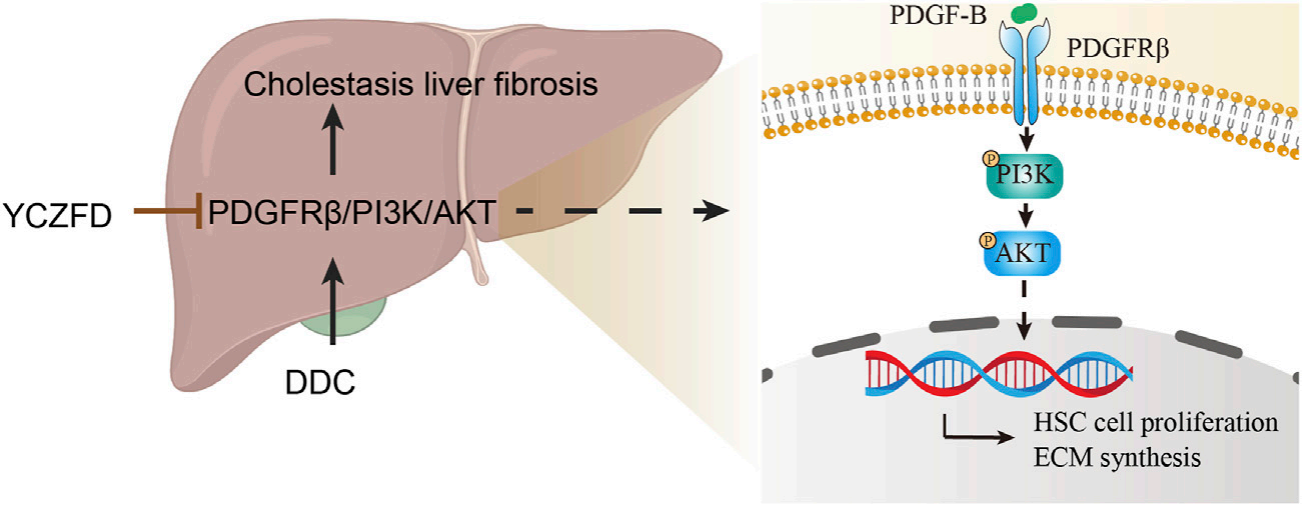
Hypothetical mechanism of action of YCZFD while protecting against hepatic fibrosis induced by DDC via the PDGFRβ/PI3K/AKT signaling pathway. YCZFD downregulated the DDC-induced increase in PDGFRβ expression. Consequently, the downregulation of PDGFRβ inhibited the PI3K-AKT signaling pathway, effectively preventing HSC cell proliferation and collagen production.

To validate the proteomics results, the protein and mRNA expression levels in the PDGFRβ/PI3K/AKT pathway in DDC-induced mice were measured through Western blot and real-time qPCR, respectively. PDGF-B may promote fibrosis in animal models and at the cellular level (Wang et al., 2016). In the present study, the protein levels of PDGFRβ/PI3K/AKT and PDGF-B were upregulated in the DDC-induced cholestatic fibrotic mice and downregulated after YCZFD treatment. Further, we conducted *in vitro* experiments in TGF-β-induced liver stellate cells HSCs (LX2 cells) because PDGFRβ is lowly expressed in quiescent HSCs but highly expressed in activated states (Ikeda et al., 1999; Bonner, 2004). In line with the *in vivo* experiment, the increased protein expression levels of PDGFRβ, PI3K, AKT, and PDGF-B induced by TGF-β were also attenuated by YCZFD. In addition, PCNA, a cofactor of DNA polymerase, is extensively expressed in the nuclei of proliferating hepatocytes. The PI3K/AKT pathway is involved in the regulation of cell cycle progression and PCNA levels and activity (Chang et al., 2003; Strzalka and Ziemienowicz, 2011). In the present study, the expression of PCNA was upregulated in TGF-β-induced LX2 cells and downregulated after YCZFD intervention. These results showed that YCZFD can reverse TGF-β-induced HSC proliferation caused by TGF-β. To investigate the mechanism by which YCZFD inhibits HSC proliferation, we analyzed the cell cycle progression of LX2 cells through flow cytometry. The results demonstrated that YCZFD inhibited LX2 growth by arresting the cell cycle in the G2/M phase. Therefore, YCZFD ameliorated CLF by inhibiting HSC proliferation and collagen production, which is associated with its role in inhibiting the PDGFRβ/PI3K/AKT signaling pathway (Figure 8). However, in the future, experiments involving PDGFRβ knockdown or overexpression are still needed to verify the effect of YCZFD on this pathway.

In addition to the findings of the present study, our previous study found that YCZFD can ameliorate chronic cholestasis by improving the homeostasis of disordered BAs and inhibiting TLR4/NF-κB-mediated inflammation of the liver tissues of mice (Li et al., 2023). This phenomenon possibly contributed to the alleviation of YCZFD on CLF because BA disorders and inflammation are also involved in the occurrence and development of CLF (Li et al., 2019a; Zhuang et al., 2023). However, the relationship between the c and PDGFRβ/PI3K/AKT pathways in cholestatic liver fibrosis warrants further research.

Moreover, according to the main plant metabolites determined as shown in Supplementary Table S1, the effects of those plant metabolites of YCZFD on PDGFRβ were further investigated in mouse primary hepatocytes. Among these plant metabolites, chlorogenic acid, scoparone, isoliquiritigenin, glycyrrhetinic acid, formononetin, atractylenolide I, and benzoylaconitine exhibited inhibitory effects on *PDGFRβ* mRNA expression. Previous studies reported that chlorogenic acid, scoparone, liquiritigenin, and glycyrrhetinic acid exhibit hepatoprotective effects against liver fibrosis (Hui et al., 2020; Wang et al., 2021c; Miao et al., 2022; Pan et al., 2022). These active plant metabolites may contribute to the protective effect of YCZFD against DDC-induced CLF by regulating PDGFRβ.

Additionally, disordered energy metabolic processes such as steroid biosynthesis, retinol metabolism, cholesterol biosynthetic processes, glutathione metabolism, arginine biosynthesis, and lipid metabolic processes participate in the development of fibrosis (Chang and Yang, 2019). Results of our proteomic analysis indicated that DEPs in Cluster 2 and Cluster 4 were significantly enriched in these metabolic processes, suggesting that YCZFD intervention can improve liver metabolic functions in cholestatic mice. However, further evaluation and validation in the future are warranted. Moreover, our previous study indicated that YCZFD can ameliorate CLI by improving the homeostasis of disordered BAs. In line with our previous study, the results of proteomic analysis in the present study showed that YCZFD increased the expression of BA metabolic enzymes, such as CYP2B10 and UGT1A1, which promoted BA metabolism. However, the influence of YCZFD treatment on the relationship between BA homeostasis and PDGFRB/PI3K/Akt pathway needs further research.

## 5 Conclusion

Our results indicate that YCZFD ameliorates CLF mainly through inhibiting the PDGFRβ/PI3K/AKT pathway. These findings support the beneficial effects of YCZFD against CLF and provide therapeutic targets for further research.

## Data Availability

The datasets presented in this study can be found in online repositories. The names of the repository/repositories and accession number(s) can be found in the article/Supplementary Material.
