# Supplementary material for: Quantitative proteomics reveals the protective effects of Yinchenzhufu decoction against cholestatic liver fibrosis in mice by inhibiting the PDGFRβ/PI3K/AKT pathway
Source: Front Pharmacol. 2024 Feb 26;15:1341020. doi: 10.3389/fphar.2024.1341020 (PMC10926276; doi:10.3389/fphar.2024.1341020)
Supplement: Supplementary file 2 [file Table2.DOCX]

**Quantitative proteomics reveals the protective effects of Yinchenzhufu decoction**

**against cholestatic liver fibrosis in mice by inhibiting PDGFRβ/PI3K/AKT pathway**

Qian Meng^1, 2^, Hongwen Zhu^2^, Yuanyuan Li^1^, Xiaotian Peng^1^, Tianming Wang^1^, Hui Huang^2^, Hu Zhou ^2, 3^, Jiayue Liu^2,4^, Sujie Ru^2,4^, Jiasheng Wu^1^*, Yueming Ma^1^ *

^1^Department of Pharmacology, School of Pharmacy, Shanghai University of Traditional Chinese Medicine, 1200 Cailun Road, Shanghai 201203, China

^2^Analytical Research Center for Organic and Biological Molecules, State Key Laboratory of Drug Research, Shanghai Institute of Materia Medica, Chinese Academy of Sciences, 555 Zuchongzhi Road, Shanghai 201203.

^3^University of Chinese Academy of Sciences, Number 19A Yuquan Road, Beijing 100049, China.

^4^School of Chinese Materia Medica, Nanjing University of Chinese Medicine, 138 Xianlin Avenue, Nanjing, Jiangsu, 210023, China

**Correspondence**

Jiasheng Wu and Yueming Ma,

Department of Pharmacology, School of Pharmacy, Shanghai University of Traditional Chinese Medicine, 1200 Cailun Road, Shanghai 201203, E-mail addresses: wujiasheng@shutcm.edu.cn (J.-S. Wu), mayueming_117@hotmail.com (Y.-M. Ma).

**Supplementary Information**

***M1：Chemicals and reagents***

Sodium dodecylsulfate (SDS), 1, 4-dicenylcel glycol (DTT), indolelactic acid, urea, ammonium bicarbonate, and trifluoro-acetate, were obtained from Sigma-Aldrich (St. Louis, MO, USA). Formic acid was purchased from Tokyo Chemical Industry (F0654; Tokyo, Japan). REPROSIL-PUR C_18_-AQ (3 μm) was purchased from Dr. Maisch GmbH (Ammerbuch, Germany). Acetonitrile (ACN) was obtained from Thermo Fisher (A955-4; Waltham, MA, USA). Dulbecco's modified Eagle medium (DMEM) and fetal bovine serum (FBS) were purchased from Gibco/BRL Life Technologies (Waltham, MA, USA). The ECL system and Amersham Hyperfilm ECL were obtained from GE Healthcare (Amersham, UK). The 10 kDa cut-off Centricon filter was obtained from Millipore (Billerica, MA, USA). The six crude drugs, including Artemisiae Scopariae Herba (*Artemisia capillaris* Thunb*.*210218), Atractylodis Macrocephalae Rhizoma (*Atractylodes macrocephala* Koidz.210430), Zingiberis Rhizoma (*Zingiber officinale* Rosc.210403), Aconiti Lateralis Radix Praeparaia (*Aconitum carmichaelii* Debx.210309), Glycyrrhizae Radix et Rhizoma Praeparata (*Glycyrrhiza uralensis* Fisch.2106034) and Cinnamomi Cortex (*Cinnamomum cassia* Presl.210309), were purchased from Shanghai Kangqiao Chinese Medicine Tablet Co., LTD; and were authenticated by Dr. Jinrong Wu from Shanghai University of Traditional Chinese medicine.

***M2:*** ***Serum Biochemistry and Histological Analysis***

Serum alanine aminotransferase (ALT), aspartate aminotransferase (AST), alkaline phosphatase (ALP), total bile acid (TBA), and total bilirubin (TBIL) levels were measured using a Hitachi 7080 Chemistry Analyzer (Hitachi Ltd., Tokyo, Japan). Liver samples were fixed in 10% formaldehyde for hematoxylin-eosin (H&E) and picrosirius red (PSR) staining.

***M3:*** ***FASP procedure***

A total of 100 μg of protein was loaded into a 10 kDa centrifugal filter tube (Millipore), washed twice with 200 μL of UA buffer (8 M urea in 0.1 M Tris-HCl, pH 8.5), alkylated with 50 mM iodoacetamide in 200 μL of UA buffer for 30 min in darkness, washed three times with 100 μL of UA buffer, followed by three additional washes with 100 μL of 50 mM NH_4_HCO_3_. After each of step, the samples were centrifuged at 12,000 *g* at 25 °C. The proteins were then digested using trypsin (Promega, Madison, WI, USA) at an enzyme-to-substrate ratio of 1:50 (w/w) in 200 μL of 50 mM NH_4_HCO_3_ at 37 °C for 16 h, and the resulting peptides were eluted through centrifugation. After digestion, peptides were subjected to desalting through C18 Stage Tips and subsequently evaporated to complete dryness using a Speed-Vac sample concentrator. Lastly, the quantities of purified peptides were assessed using a Nano Drop instrument (Thermo Fisher).

***M4: Real-Time PCR***

Total RNA was extracted from liver tissues or LX-2 cells using TRIzol (TAKARA, Kusatsu, Japan) according to the manufacturer’s protocol. Total RNA with a 260/280 nm ratio of > 1.8 and 260/230 nm ratio of > 2.0, as measured using a NanoDrop spectrophotometer (Thermo Fisher), was used in subsequent PCR analyses. A High Capacity cDNA Reverse Transcription Kit (TAKARA) was employed to synthesize cDNA and an SYBR-Green Universal Master Mix Kit was employed to quantitatively evaluate gene expression levels using the 7500 Fast Real-Time PCR system (Applied Biosystems, Irvine, CA, USA). Relative mRNA expression was calculated using the 2^−ΔΔCt^ method. The primer sequences (Table S3) were purchased from Shanghai Generay Biotech Co., Ltd. (Shanghai, China).

***M5: Western Blot Analysis***

Mouse liver tissues or LX-2 cells were lysed with 4% SDS lysis buffer containing 0.1M DTT. Protein lysates were separated by SDS-PAGE and transferred to polyvinylidene fluoride (PVDF) membranes. Then, PVDF) membranes were blocked with 5% BSA at 25 ℃ for 1 h and incubated with primary antibodies at 4 ℃ overnight. After three washes in TBST, membranes were incubated with a goat anti-rabbit secondary antibody (ABclonal) for 1 h at room temperature. Then, membranes were scanned using ImagQuant (GE Healthcare). The experiment was repeated at least three times.

**Supplementary Tables**

Supplementary table 1. Identiﬁed compounds in Yinchenzhufu Decoction (YCZFD)

Supplementary table 2. Content determination in YCZFD extract.

Supplementary table 3. Gene sequence of primers.

|  |  |
| --- | --- |

**Figure S1**


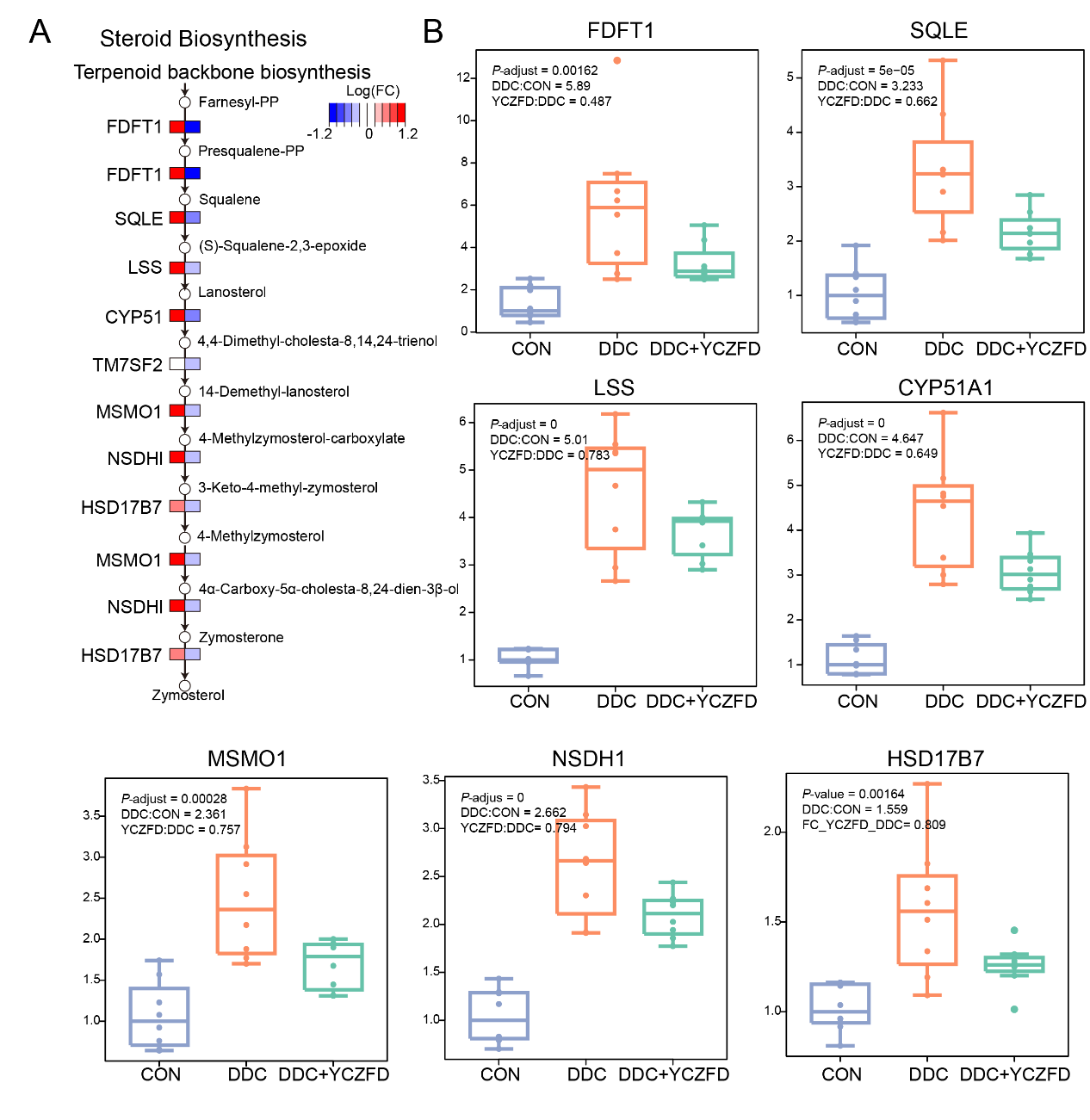


Figure S1 Sterol metabolism pathway and box-plot of related proteins' expression level.

**Figure S2**


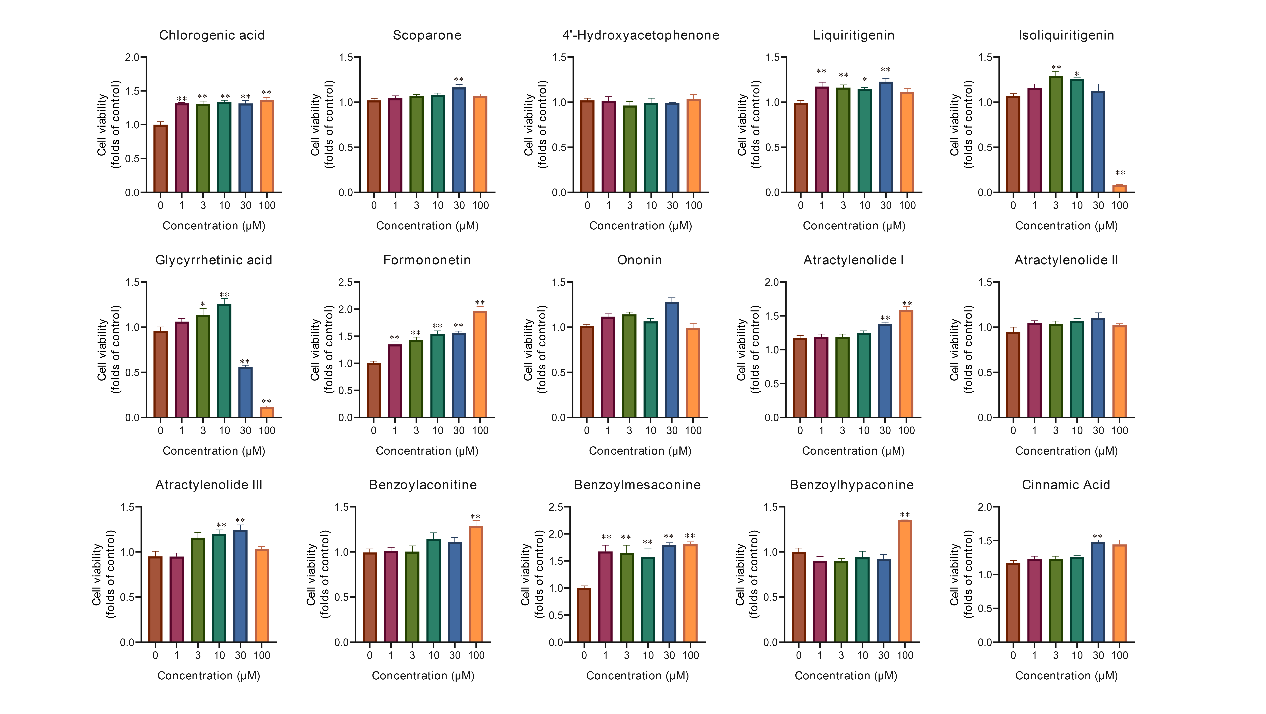


Figure S2 The results of the CCK-8 assay provide information about the drug's impact on cell health at different concentrations (n=3). Data are presents by means ± SD; **p < 0.01 and *p < 0.05 compared with control group.
